# A Sensing Peak Identification Method for Fiber Extrinsic Fabry–Perot Interferometric Refractive Index Sensing

**DOI:** 10.3390/s19010096

**Published:** 2018-12-28

**Authors:** Bowen Yang, Biyao Yang, Ji Zhang, Yiheng Yin, Yanxiong Niu, Ming Ding

**Affiliations:** School of Instrumentation and Optoelectronic Engineering, Beihang University, No. 37 Xueyuan Road, Haidian District, Beijing 100083, China; yangbw@buaa.edu.cn (B.Y.); yangbiyao@buaa.edu.cn (B.Y.); jizhang@buaa.edu.cn (J.Z.); yinyh1203@163.com (Y.Y.); niuyx@buaa.edu.cn (Y.N.)

**Keywords:** sensing peak identification, Fabry–Perot interferometer, optical fiber sensor, refractive index sensing

## Abstract

A novel sensing peak identification method for high accuracy refractive index (RI) sensing is proposed. The implementation takes the intensity of interference maximum as the characteristic to distinguish interference peaks, tracking the sensing peak continually during a RI changes, with high measurement accuracy and simple computation. To verify the effect of the method, the extrinsic Fabry–Perot interferometer (EFPI) sensor has been fabricated using the large lateral offset splicing technique. In the RI range from 1.346 to 1.388, the measurement range of the EFPI with the proposed method reaches at least 6 times larger than that of EFPI with the wavelength tracking method and the largest measurement error is −4.47 × 10^−4^. The EFPI refractive index (RI) sensor identified the sensing peak is believed to play an important role in RI, concentration and density sensing, etc., for superior performance.

## 1. Introduction

With excellent performance in measurement accuracy, resolution, and stability, the Fabry–Perot fiber interferometer (FPI) has attracted tremendous interest for use in temperature [1,2], pressure [3,4], strain [5,6] sensing, biosensing [7,8], and dispersion measuring [9] applications. The FPI can be classified as intrinsic Fabry–Perot interferometer (IFPI) or extrinsic Fabry–Perot interferometer (EFPI) according to their construction. Comparing to the IFPI, the cavity of EFPI can be opened to the environment. It expands the sensing application to a larger area such as refractive index (RI) [10,11], density [12], and concentration [13] which can be used to monitor the battery state-of-charge, water pollution, air quality, and so on. 

Since the optical spectrum analyzer (OSA) was applied to the EFPI sensor demodulation, an algorithm has always been necessary for extracting effective information from the interference spectrum. The most influential demodulation algorithm is the wavelength tracking method [14], because of the advantages of high resolution and simple calculation. However, it is also well-known that this demodulation algorithm has a stubborn problem named the fringe direction ambiguity which obstructs the pervasive practical application of the EFPI sensor [15]. When the spectrum drifts over half the free spectrum range (FSR), the drift direction of the interference peaks becomes ambiguous due to the periodicity of the Fabry–Perot interference spectrum. Therefore, the dynamic range of the EFPI sensor based on the wavelength tracking method is limited to less than half FSR, resulting in limitation of the measurement range and impairment to the utility values. Numerous demodulation algorithms have been reported to identify the sensing peak of EFPI sensors. A method that calculates the optical path difference (OPD) between two adjacent peak wavelengths in the spectrum was proposed [16]. The dynamic range of EFPI sensors could be enlarged greatly, while the resolution became deficient [17,18]. The low resolution would impair the accuracy of the calculated OPD, meaning that the accuracy of measured physical quantities is limited with this method. B. Qi et al. improved the wavelength tracking method by involving the adjacent peak of the tracked peak, ascertaining the interference order number before calculating the OPD [19]. Although the interference spectrum of EFPI is periodic, the interference peaks can also be distinguished by the interference order, which is unique for each peak in the spectrum. However, the interference order calculation may obtain a wrong result due to integer rounding error [20]. G. Liu et al. proposed an improved method to reduce the integer rounding error using two far-separated peaks in the spectrum [15]. This method has an ideal experiment result, whereas the algorithm is highly complicated which may dramatically slow down the computation speed. T. Liu et al. applied the Fourier transform on the interference spectrum to avoid the fringe direction ambiguity; nevertheless, the basic frequency is insensitive to OPD changes [21]. To improve the sensitivity of the basic frequency, Y. Jiang distracted the phase information based on the band-pass filter and Fourier inversion [22]. The OPD sensitivity was enhanced while the calculating process became much more complicated. A cross-correlation method, which calculates the cross-correlation between the actual interference spectrum and a simulated interference spectrum with changed OPD, was proposed [23]. The simulated OPD can be taken as the measured result when the cross-correlation reach its maximum. This method achieved high measurement accuracy, accompanied by lower calculating speed.

In this paper, a novel sensing peak identification method for high accuracy RI sensing is proposed. It takes advantage of the relationship between the interference maximum intensity and the sensing peak wavelength to distinguish the sensing peak from the interference spectrum. This method is theoretically proved to be effective based on simple calculation. The experimental verification is implemented through use of an EFPI refractive index (RI) sensor based on a large lateral offset splicing structure. It shows that the measurement range of the EFPI RI sensor is several times larger than that of wavelength tracking method and the RI measuring error is quite small.

## 2. Principle of Operation

**Figure 1:**
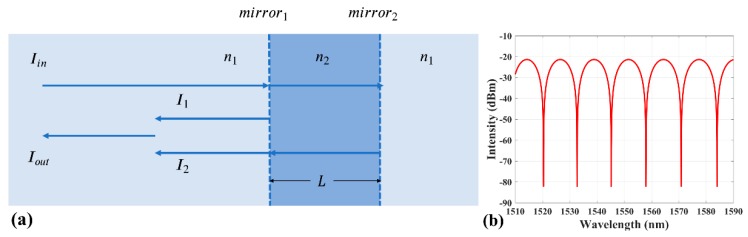
(**a**) The schematic diagram of Fabry–Perot interferometer (EFPI) sensor. (**b**) The theoretical reflection spectrum of EFPI refractive index (RI) sensor.

The schematic diagram of fiber EFPI RI sensor is shown in Figure 1a. The construction has two mirrors and three areas of medium with two different refractive indices *n*_1_ and *n*_2_. The distance between the two mirrors is *L*. As shown in Figure 1, when an incident light beam with intensity *I*_in_ is transmitted into the fiber core, Fresnel reflections take place on the two mirrors, resulting in two reflection beams with intensities *I*_1_ = *I*_in_*R*_1_ and *I*_2_ = *I*_in_*T*_1_*R*_2_*T*_2_, where *R*_1_, *T*_1_, *R*_2_, and *T*_2_ represent the reflectance and transmittance of the two mirrors, respectively. Under the assumption that the medium on both sides of the Fabry–Perot (FP) cavity have the same refractive index, the reflectance and transmittance can be simplified as $R_{1}=R_{2}={\left(\frac{n_{1}-n_{2}}{n_{1}+n_{2}}\right)}^{2}$ and $T_{1}=T_{2}=\frac{4n_{1}n_{2}}{{\left(n_{1}+n_{2}\right)}^{2}}$.

The interference can be simplified as occurring between two beams because of the low mirror reflection. Therefore, the intensity of output light can be derived by

(1) $$I_{out}=I_{1}+I_{2}+2\sqrt{I_{1}I_{2}}\cos\delta =I_{in}(R_{1}+R_{1}T_{1}{}^{2}+2R_{1}T_{1}\cos\delta ),$$

where the half-wave loss has been ignored and the phase difference is $\delta =\frac{4\pi n_{2}L}{\lambda }$. Figure 1b shows the theoretical reflection spectrum of EFPI RI sensor according to the Equation (1). The intensity of interference maximum can be calculated by

(2) $${\left(I_{out}\right)}_{\max}=I_{in}(R_{1}+R_{1}T_{1}{}^{2}+2R_{1}T_{1})=I_{in}\frac{n_{1}^{3}-5n_{1}n_{2}^{2}+5n_{1}^{2}n_{2}-n_{2}^{3}}{{\left(n_{1}+n_{2}\right)}^{6}}.$$

The wavelength of the *k*th order of interference maximum can be expressed by

(3) $$\lambda =\frac{2n_{2}L}{k}.$$

**Figure 2:**
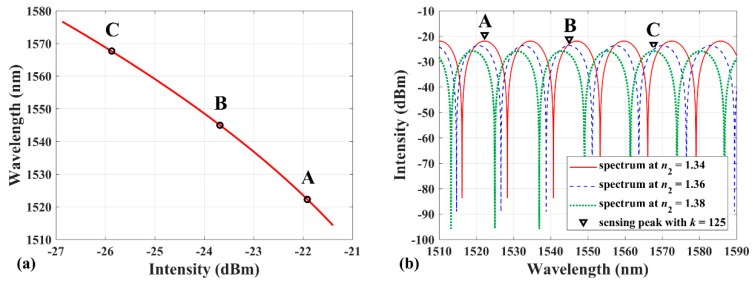
(**a**) Relationship between the sensing peak wavelength and the interference maximum intensity and (**b**) interference spectrum at *n*_2_ = 1.34, 1.36, and 1.38, respectively. The marked points A, B, and C represent the same sensing peak (*k* = 125); the parameters *L* = 71 μm, *I*_in_ = 1 mW, *n*_1_ = 1.45205; and *n*_2_ changes from 1.333 to 1.388.

From Equations (2) and (3) it can be seen that the wavelength of sensing peak (with specific interference order) and the intensity of interference maximum are both related to the RI *n*_2_. Eliminating the RI *n*_2_ in Equations (2) and (3) can derive the relationship between the interference maximum intensity and the sensing peak wavelength, called the location curve. Therefore, the sensing peak wavelengths at different RI environments can be located by the interference maximum intensity. Figure 2 exhibits the theoretical method to locate the sensing peak when the spectrum drift over half FSR, in which *I*_in_ = 1 mW, *n*_1_ = 1.45205, *L* = 71 μm, and *k* = 125. The parameters are decided by the EFPI sensor and the bandwidth of light source used in the following experiment. Figure 2a depicts the monotone decreasing relation of the location curve, in which *n*_2_ changes from 1.333 to 1.388. Figure 2b shows the interference spectrums at *n*_2_ = 1.34, 1.36, and 1.38, respectively. The three points marked by A, B, and C in Figure 2 represent the sensing peak (with *k* = 125) at three different RI environments. It is obvious that the sensing peak is recognized successfully by the location curve even though the spectrum drift is over half FSR. The intensity of interference maximum becomes a characteristic of the wavelength of sensing peak, which can be used to distinguish the sensing peak from the interference spectrum. Compared with the interference order, the interference maximum intensity is easy to obtain without integer rounding error. 

Due to the measuring and calculating error, there is a slight difference between the calculated sensing peak wavelength and the actual sensing peak wavelength. The difference is much less than half FSR, therefore in the actual interference spectrum, the nearest peak from the calculated sensing peak can be taken as the actual sensing peak. The calculated sensing peak is named as rough sensing peak. 

Therefore, a novel sensing peak identification method for RI sensing can be realized by combining the location curve and the relationship between the sensing peak wavelength and the RI of environment (response curve). Applying this method has the advantages of high measurement accuracy and is undisturbed to the light source fluctuation compared to calculating the RI of environment by the interference maximum intensity directly. 

**Figure 3:**
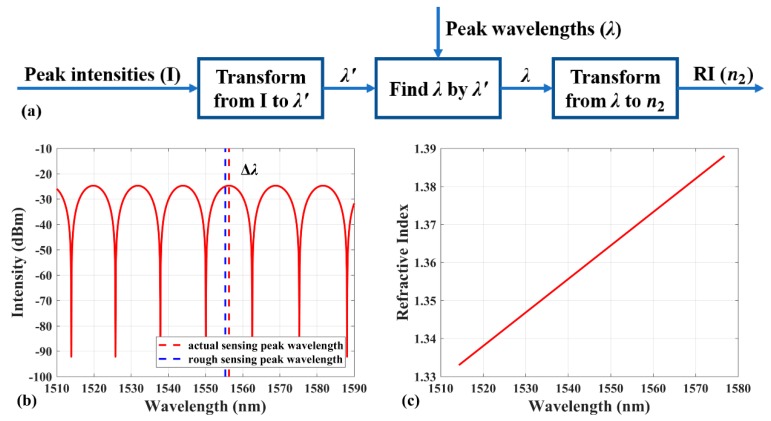
(**a**) Block flow diagram of the total demodulation process. (**b**) The schematic diagram of finding actual sensing peak by the rough sensing peak. (**c**) The theoretical response curve of EFPI RI sensor.

Figure 3a presents the block flow diagram of the total demodulation process. The computational procedure can be divided to three steps. First, using the interference maximum intensity *I* to calculate the rough sensing peak wavelength λ’ according to the location curve. Second, the actual sensing peak is obtained by searching the nearest peak from the rough sensing peak in the interference spectrum. As shown in Figure 3b, only if Δλ is less than half FSR of the interference spectrum, the nearest actual interference peak, whose wavelength is λ, can be taken as the actual sensing peak. The length of FP cavity can be adjusted to ensure that the half FSR is larger than Δλ. The combination of the two steps is named as the location process for the destination of locating the actual sensing peak at arbitrary RI environment. The last step is to calculate the RI by the actual sensing peak wavelength λ according to the response curve depicted in Figure 3c. 

The sensing peak identification method consisted of the former two steps can be processed automatically with very simple calculation. Furthermore, this demodulation has high accuracy thanks to the high resolution of interferometry and the measurement range, which includes arbitrary RI except a small section around the RI of the fiber core theoretically. 

## 3. Sensor Fabrication and Experimental Results

### 3.1. Sensor Fabrication

**Figure 4:**
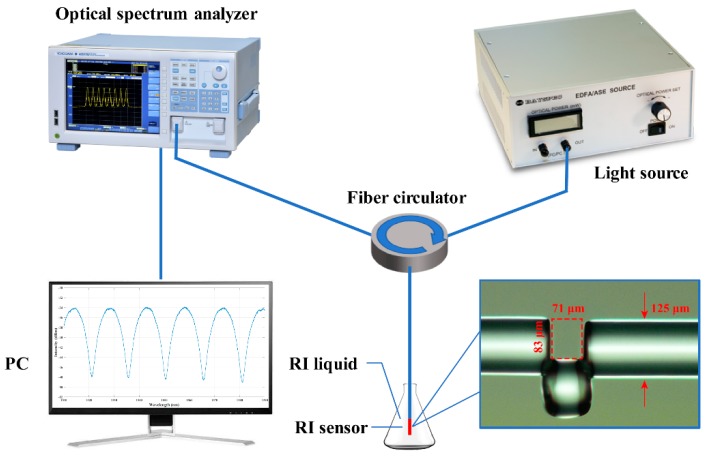
Experimental setup for liquid refractive index sensing. Insert, the micrograph of EFPI sensor.

The fiber EFPI RI sensor fabricated by the large lateral offset splicing technique was utilized to verify the proposed method of sensing peak identification. The insert in Figure 4 represents the EFPI sensor microscope image, which consists of three sections of single mode fiber (SMF) with identical materials (SMF-28, Corning, New York, NY, USA). The junctions between the three sections of fiber are spliced using the fiber fusion splicer (S178C, FITEL, Tokyo, Japan). The FP cavity length is 71 μm and the displacement of lateral offset is 83 μm which is a compromise between the firmness of sensor and flatness of mirrors. The end of the fiber EFPI sensor is angle cleaved by 8 degrees to avoid the end reflection. 

### 3.2. Experimental Setup

The liquid RI sensing experimental setup is shown in Figure 4, including the fiber EFPI RI sensor, an OSA (AQ6370D, YOKOGAWA, Tokyo, Japan), a broadband light source from 1510–1590 nm (MiniLite, Bayspec, San Jose, CA, USA), a fiber circulator, RI liquids, and a computer. The interference peak wavelengths and intensities can be extracted by computer. 

### 3.3. Calibration

**Figure 5:**
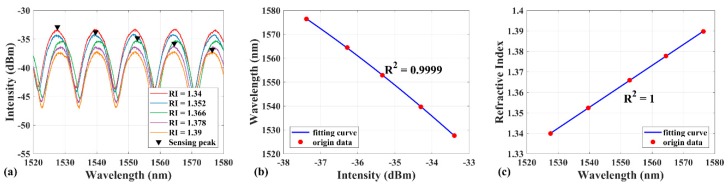
(**a**) The actual interference spectrum with five different RI liquids; the peaks marked with the black triangle are the actual sensing peaks with same interference order. (**b**) Location curve and (**c**) response curve of the EFPI RI sensor. The red scatter points and blue curves represent the experimental data and the fitting curves separately.

Before verifying the sensing peak identification method, the fiber EFPI RI sensor was calibrated first. Five bottles of liquids with different known RI from 1.34 to 1.39 were selected to calibrate the sensor, with the purpose of covering the wavelength range of the light source. The interference maximum wavelengths and intensities were recorded when the sensor head was inserted into different bottles of RI liquids, respectively. Before inserting a different RI liquid, the sensor head was cleaned with ethanol and deionized water to ensure that the sensor head did not have residue of the former RI liquid. Figure 5a exhibits the actual interference spectrum at 5 bottles of RI liquids, in which the 5 points marked with black triangle represent the actual sensing peak. The calibration process was designed and verified in advance. Therefore, the actual sensing peak in spectrum would hardly be selected to the wrong peak. Figure 5b,c presents the location curve and the response curve of the sensor, which are found to be mostly in agreement with the theoretical simulation (Figure 2a and Figure 3c). The data points marked in Figure 5a are fitted by a second-order polynomial to obtain the location curve, in which R^2^ = 0.9999. Similarly, the sensing peak wavelength in Figure 5a and the RI of liquids were fitted by linear fitting to obtain the response curve, in which R^2^ = 1. It also reveals the RI sensitivity is 979.7 nm/RIU. 

### 3.4. Verification

**Figure 6:**
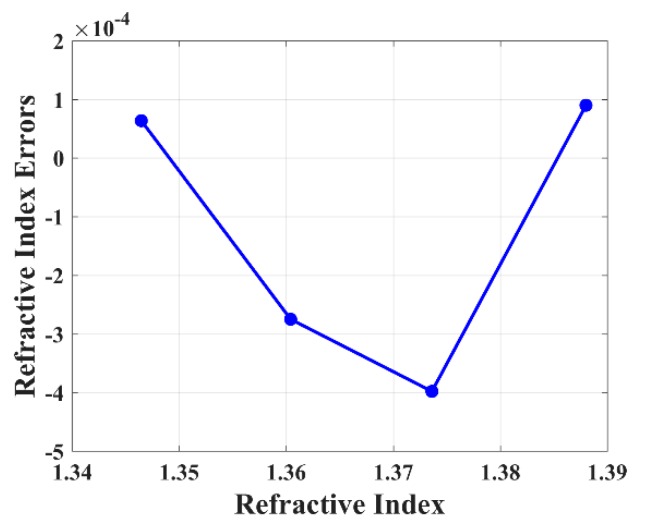
The measurement errors in five different RI liquids.

The sensing peak identification method was verified by immersing the EFPI RI sensor into another 4 bottles of liquid with different RI from 1.346 to 1.388. As the input data of the method, the interference maximum wavelengths and intensities were recorded so that the liquid RI could be calculated automatically through the proposed algorithm. Figure 6 exhibits that the measurement errors, which is defined as the difference between the calculated liquid RI and standard liquid RI, are all less than −4 × 10^−4^. The small RI error reveals the high accuracy of the EFPI RI sensor attributed to the superiority of interferometry. On the other hand, it indicates that the location process runs well, for the reason that if the wavelength error were over the half FSR of interference spectrum, the adjacent peak would be taken as the sensing peak by mistake. It would lead to a RI error over 0.0134, which is two orders of magnitude larger than the RI error in Figure 6. Moreover, the RI measurement range reaches 0.042 applying the proposed method, which is at least 6 times larger than that calculated by the wavelength tracking method under the same conditions (0.0067 according to the half FSR limitation). The RI range from 1.346 to 1.388 in the experiment is determined by the broadband light source from 1510 to 1590 nm. The sensing peak would drift out of the spectrum if the RI range became wider without a broader light source. If the broader bandwidth of light source were utilized in the experiment, the EFPI RI sensor would reach a larger RI range.

## 4. Discussion

**Figure 7:**
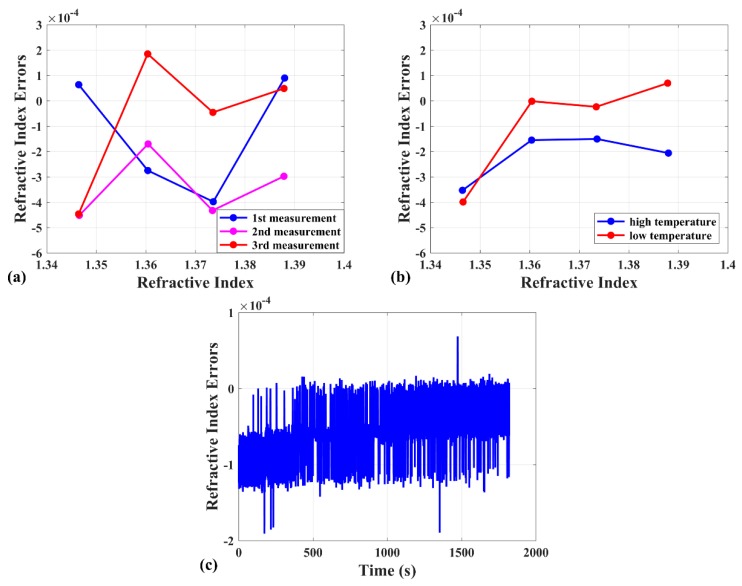
(**a**) The RI errors at three times of the repeating measurement. (**b**) The RI errors at two times of the temperature stability test. (**c**) The RI errors changes in 30 min in the RI = 1.36 liquid.

The sensing peak identification method for the EFPI RI sensor is part of the demodulation based on the interference peak wavelengths and intensities. It is a universal calculation method that can be applied to different kinds of liquid or gas RI sensing. The structure other than lateral offset splicing structure to constitute EFPI is allowed as well. The repeatability, temperature stability, and long-time stability of the EFPI RI sensor with the proposed method were tested. Figure 7a exhibits the RI errors at three times repeating measurement under the same conditions, in which the largest RI error is −4.47 × 10^−4^. Figure 7b presents that the largest RI error is −4 × 10^−4^ at two times of temperature stability test. The temperature stability test was carried out in a thermostat. For the high temperature part, the thermostat was set to 75 °C for an hour and slowly cooled to room temperature. Regarding the low temperature part, it was set to −25 °C with the same process. Figure 7c reveals the stability of the sensing peak identification method in 30 min. The result shows the changes of RI errors is 1.57 × 10^−5^ and the range is 2.58 × 10^−4^. The abnormal distribution of RI errors is caused by the algorithm of extracting the interference peak wavelengths while attempting optimization. Moreover, an obviously ascending tendency for the RI errors changes can be observed which is resulted by the evaporation of water in RI liquid. 

The sensitivities and ranges of different sensor constructions are compared in Table 1. It can be observed that the range of EFPI RI sensor with the sensing peak identification method in this work is superior to other constructions, though the sensitivity is not prominent. 

## 5. Conclusions

In summary, a novel sensing peak identification method has been proposed. The experimental result shows the measurement range from 1.346 to 1.388, which is at least 6 times larger than that of the wavelength tracking method. The largest measurement errors were −4.47 × 10^−4^ and −4 × 10^−4^ with triplicate repeating measurements and duplicate temperature stability tests, respectively. The stability of this solution has been proved according to a 30 min continuous measurement in which the variation of RI errors is 1.57 × 10^−5^ and the range is 2.58 × 10^−4^. The EFPI RI sensor identified the sensing peak has the advantages of large measurement range and high accuracy. It is anticipated that the enhanced RI sensor can be applied to in liquid or gas RI, concentration or density monitoring, and state-of-charge detection, pollution monitoring, and so on.

## Figures and Tables

**Table 1:** Sensitivities and ranges of different sensor constructions.

Sensors	Sensitivity (nm/RIU)	Range (RIU)
Micro-tapered long-period fiber grating [24]	8188.1	1.332–1.3328
Open-cavity Mach–Zehnder interferometer [25]	−1364.343	1.333–1.3468
Inline Mach–Zehnder interferometer coated with hafnium oxide [26]	1307	1.3327–1.3478
Gold-coated side-polished photonic crystal fiber [27]	−40400	1.415–1.42
EFPI RI sensor (This work)	979.7	1.346–1.388
